# Imeglimin as a Replacement Therapy for Metformin in Patients With Type 2 Diabetes Mellitus Experiencing Metformin Intolerance: A Retrospective Observational Study

**DOI:** 10.7759/cureus.110200

**Published:** 2026-06-03

**Authors:** Debmalya Sanyal, Rituraj B Deb

**Affiliations:** 1 School of Medicine and Public Health, The Newcastle University, Newcastle, AUS; 2 Endocrinology, Narayana Health Rabindranath Tagore International Institute of Cardiac Sciences (NH RTIICS), Kolkata, IND; 3 Endocrinology, Kali Prasad Chowdhury (KPC) Medical College and Hospital, Kolkata, IND; 4 Pharmacology, Zydus Healthcare Limited, Mumbai, IND

**Keywords:** glycemic control, imeglimin, metformin intolerance, mitochondrial modulation, replacement therapy, type 2 diabetes mellitus

## Abstract

Background

Type 2 diabetes mellitus (T2DM) is a major global health burden. Despite being the preferred first-line therapy, metformin use is sometimes limited by gastrointestinal intolerance, creating a need for alternative management strategies. A promising oral antidiabetic substitute, imeglimin, a first-in-class tetrahydrotriazine, has a distinct mitochondrial mechanism of action. However, its real-world efficacy and tolerability as a direct replacement for metformin in GI-intolerant patients remain uncharacterized.

Methods

This retrospective observational study was conducted on 100 adult patients with T2DM who had documented metformin intolerance and were initiated on imeglimin monotherapy or combination therapy for three months. Primary outcomes included variations in glycated hemoglobin (HbA1c), fasting plasma glucose (FPG), and postprandial plasma glucose (PPG). Body weight, lipid profile, and renal function were the secondary outcomes. Normality was assessed using the Shapiro-Wilk test; paired t-tests and Wilcoxon signed-rank tests were applied as appropriate. Multivariate linear regression was performed with change in HbA1c as the dependent variable and key covariates (baseline HbA1c, age, sex, T2DM duration, body weight, imeglimin dose, and number of concomitant oral anti-diabetic drugs) as independent variables. A two-tailed p<0.05 was considered statistically significant.

Results

The study comprised 100 patients (55% female; mean age: 57.69 ± 9.24 years; mean T2DM duration: 10.38 ± 5.69 years). HbA1c decreased significantly from 7.96 ± 1.56% to 7.35 ± 1.02% (mean change: −0.62 ± 1.05%; p<0.001; n=77) after three months of imeglimin therapy. FPG decreased from 156.63 ± 48.48 mg/dL to 124.45 ± 20.46 mg/dL (−32.18 ± 47.02 mg/dL; p<0.001), and PPG decreased from 217.85 ± 84.08 mg/dL to 173.22 ± 39.71 mg/dL (−44.63 ± 68.44 mg/dL; p<0.001). Body weight decreased significantly (−0.81 kg; p<0.001). Low-density lipoprotein cholesterol and triglyceride levels also showed significant reductions (p<0.001). The estimated glomerular filtration rate remained stable (p=0.135), and gastrointestinal side effects were reported in only 2% of the patients.

Conclusion

Imeglimin demonstrated meaningful improvements in glycemic control, with a favorable safety and tolerability profile in metformin-intolerant patients with T2DM. These findings support the use of imeglimin as an effective and well-tolerated alternative option for metformin in patients with T2DM.

## Introduction

Type 2 diabetes mellitus (T2DM) is a global health burden, affecting 537 million adults worldwide aged 20-79 years in 2021 and projected to rise to 783 million by 2045 [1]. India is ranked as the second-largest diabetic population in the world and has an estimated 101 million patients with T2DM in 2023 [2]. This epidemic has a profound burden of microvascular and macrovascular complications, premature mortality, and increased healthcare expenditure in both high-income and lower-middle-income countries [1]. Glycemic control plays a key role in the prevention of the onset and progression of complications, and pharmacological therapy is essential for the majority of patients.

Metformin, which is a biguanide, lowers glycemic levels by inhibiting hepatic gluconeogenesis through suppression of mitochondrial complex I activity and subsequent activation of adenosine monophosphate-activated protein kinase [3]. For decades, major international guidelines such as the American Diabetes Association (ADA), the European Association for the Study of Diabetes (EASD), and the International Diabetes Federation (IDF) have recommended metformin as a first-line pharmacological drug for treating diabetes [4,5]. The extensive clinical evidence, well-established safety profile, neutral effect on cardiovascular outcomes and body weight, and low cost have established metformin as the foundation of therapy for most patients with T2DM in the absence of contraindications. Nevertheless, gastrointestinal (GI) intolerance is a clinically significant and neglected constraint that affects sustained therapy.

Metformin exhibits various actions in the gut, such as increased intestinal glucose uptake, elevation of glucagon-like peptide-1 (GLP-1) concentrations, accumulation within intestinal cells, and altered gut microbiota, which, in combination with other factors, predispose to GI side effects in susceptible individuals [6]. Around 20%-30% of patients on metformin develop adverse effects such as nausea, vomiting, abdominal cramps, diarrhea, and bloating [7]. Permanent discontinuation of metformin occurs in ~5% of patients due to GI intolerance, even with gradual dose titration or a switch to extended-release formulations [6]. Some individuals are susceptible to these adverse effects because of organic cation transporter 1 variants and other genetic factors, which were further confirmed by pharmacogenomic studies. However, no reliable screening strategy exists in routine clinical practice [8,9]. Metformin intolerance seems to be intrinsically linked to drug retention in the intestinal wall and to drug effects on downstream serotonergic and GLP-1-mediated pathways that modulate intestinal motility [6]. There exists a clinical need for a well-tolerated alternative to metformin that maintains or improves glycemic control and does not cause an additional GI burden after permanent discontinuation of metformin.

Imeglimin (1000 mg twice daily) is the first regulatory-approved agent from Japan for the treatment of T2DM, which belongs to the tetrahydrotriazine-containing “glimin” class and has a novel pharmacological mechanism in oral antidiabetic therapy [10]. Its mechanism of action is fundamentally different from that of all other existing classes. On the cellular level, imeglimin regulates mitochondrial bioenergetics by partial/competitive inhibition of respiratory complex I while simultaneously restoring complex III activity, leading to rebalanced electron transportation, reduced production of reactive oxygen species, and prevention of mitochondrial permeability transition pore opening that drives cell death under hyperglycemic conditions [11]. Imeglimin has two complementary pharmacological effects that use these mitochondrial effects: (i) in pancreatic β-cells, it enhances glucose-stimulated insulin secretion (GSIS) by increasing mitochondrial generation of ATP and enhancing the nicotinamide adenine dinucleotide (NAD+) pool via the salvage pathway, contributing to calcium-dependent insulin granule exocytosis [12]. (ii) In hepatocytes and skeletal muscles, it restores deficient mitochondrial respiratory function, suppressing hepatic gluconeogenesis and improving insulin sensitivity through pathways that partially overlap with, but are mechanistically distinct from, those of metformin and incretin-based agents [13].

The pivotal phase III Trials of IMEglimin for Efficacy and Safety (TIMES), comprising three randomized controlled trials (RCTs), evaluated imeglimin in Japanese patients. TIMES 1 demonstrated that imeglimin 1000 mg twice daily as monotherapy in 24 weeks decreased glycated hemoglobin (HbA1c) by 0.87% compared with placebo [14]. The long-term TIMES 2 trial established extended efficacy and tolerability during 52 weeks of monotherapy and in combination with eight different antidiabetic drug classes in an open-label design [15]. TIMES 3 confirmed that the combination of imeglimin with insulin therapy resulted in a significant placebo-corrected reduction in HbA1c of 0.60% at week 16, with sustained benefit up to 52 weeks [16]. Emerging real-world data from the TWINKLE postmarket study have further demonstrated that in patients with advanced chronic kidney disease (estimated glomerular filtration rate (eGFR) < 45 mL/min/1.73 m²), imeglimin can be used safely in an adjusted-dose regimen when other antidiabetic agents are contraindicated [17].

Despite growing evidence supporting imeglimin's efficacy, a critical gap persists in the published literature: data specifically characterizing imeglimin as a direct replacement therapy in patients with established metformin intolerance remain sparse. This has become a common scenario where clinicians frequently encounter patients with heightened GI sensitivity, requiring a substitute that is not only glycemically effective but also well tolerated from a GI perspective. Imeglimin’s primary mechanism differs fundamentally from metformin's yet addresses several overlapping metabolic defects. Therefore, understanding imeglimin’s efficacy and tolerability as a substitute for metformin in GI-intolerant patients is both clinically and mechanistically important.

Therefore, we retrospectively evaluated the efficacy and safety of imeglimin as a replacement therapeutic agent in adult patients with T2DM who had GI discomfort due to metformin, over a three-month follow-up period. The primary objective was to assess changes in HbA1c, fasting plasma glucose (FPG), and postprandial plasma glucose (PPG) following the transition to imeglimin, with secondary objectives, including body weight, lipid profile, and renal and safety parameters.

## Materials and methods

Study design

This retrospective, single-center, observational study was conducted at a tertiary hospital in India between November 2023 and November 2025. Patients who were prescribed imeglimin (defined as the time of switching from metformin to imeglimin) and were followed up for three months were included in the study. Clinical and biochemical data were collected at baseline and at the end of the three-month follow-up visit.

Study population

Patients were eligible for inclusion if the following criteria were met: (i) adults aged ≥18 years with a confirmed diagnosis of T2DM previously treated with metformin; (ii) documented metformin intolerance (defined as the occurrence of persistent GI effects, including nausea, vomiting, abdominal pain, bloating, or diarrhea) as per the physician’s discretion for the diagnosis of metformin intolerance. This includes both immediate-release and extended-release formulations necessitating permanent discontinuation; (iii) initiated imeglimin as a replacement drug by the treating physician; and (iv) had available baseline and three-month follow-up data. The exclusion criteria included type 1 diabetes mellitus; severe hepatic failure, active hepatic disease (alanine transaminase or aspartate transaminase > 3× upper limit of normal at baseline); severe renal impairment (eGFR<30 mL/min/1.73m²); New York Heart Association (NYHA) class III or IV heart failure; active malignancy; pregnancy or lactation; recent hospitalization for diabetic ketoacidosis or hyperosmolar hyperglycemic state; known hypersensitivity to imeglimin; use of systemic corticosteroids; and lost to follow-up.

Intervention

Following metformin intolerance, medical records were reviewed to retrieve imeglimin dosing as prescribed by the treating physician based on individual clinical assessment, renal function, and concomitant therapy. Doses ranging from 500 mg to 2000 mg daily were documented. Concomitant oral anti-diabetic drugs (OADs) (excluding metformin) were recorded as continued at stable doses unless clinically indicated adjustments were noted. Indian published data comparing 1000 mg metformin with 1000 mg imeglimin demonstrate comparable glycemic efficacy; the dose of metformin was replaced with an equivalent dose of imeglimin (1000 mg) in patients who were previously on 1000 mg metformin. This dose-equivalence approach, supported by Indian clinical evidence, forms an important methodological basis for the replacement strategy employed in this study [18].

Assessments

Clinical and biochemical assessments were retrieved from medical records at baseline and at three months. The primary outcomes were FPG (mg/dL), PPG (mg/dL), and HbA1c (%). The secondary outcomes were changes in body weight (kg), lipid profile (low-density lipoprotein cholesterol (LDL-C; mg/dL), high-density lipoprotein cholesterol (HDL-C; mg/dL), and serum triglycerides (TGs; mg/dL), eGFR (mL/min/1.73 m²), and safety parameters, including GI adverse events. Adverse drug reactions and clinical events were retrieved from documented case records.

Statistical analysis

Continuous variables were expressed as mean ± standard deviation (SD) for normally distributed data and as median with interquartile range (IQR) for non-normally distributed data. Categorical variables were expressed as frequency and percentage. Data distribution was assessed using the Shapiro-Wilk test before statistical analysis. For within-group comparisons between baseline and three-month values, the paired samples t-test was used for normally distributed continuous variables and Wilcoxon signed-rank test for non-normally distributed variables. A two-tailed p-value <0.05 was considered statistically significant. To address potential confounding by concomitant antidiabetic medications, a multivariate linear regression analysis was performed with change in HbA1c (∆HbA1c) as the dependent variable and the following pre-specified covariates as independent variables: baseline HbA1c, age, sex, duration of T2DM, baseline body weight, imeglimin dose, and number of concomitant antidiabetic agents. All analyses were performed using SPSS version 25.0 (IBM Corporation, Armonk, NY, USA). Since this was a retrospective, real-world observational study, an a priori sample size calculation was not feasible; therefore, all eligible patients fulfilling the predefined inclusion criteria during the study period were included in the analysis. Given the observational nature of the study, residual confounding from concomitant medications and lifestyle interventions cannot be completely excluded.

Ethical consideration

The study was conducted in accordance with the Declaration of Helsinki and applicable regulatory requirements. The study protocol was reviewed and approved by the Central Independent Ethics Committee (CIEC), Pune, Maharashtra, India (CIEC/2025/04, dated 01 April 2025). In view of the retrospective nature of the study and use of de-identified medical record data, the requirement for informed consent was waived/not applicable as per ethics committee approval.

## Results

Baseline characteristics

**Figure 1:**
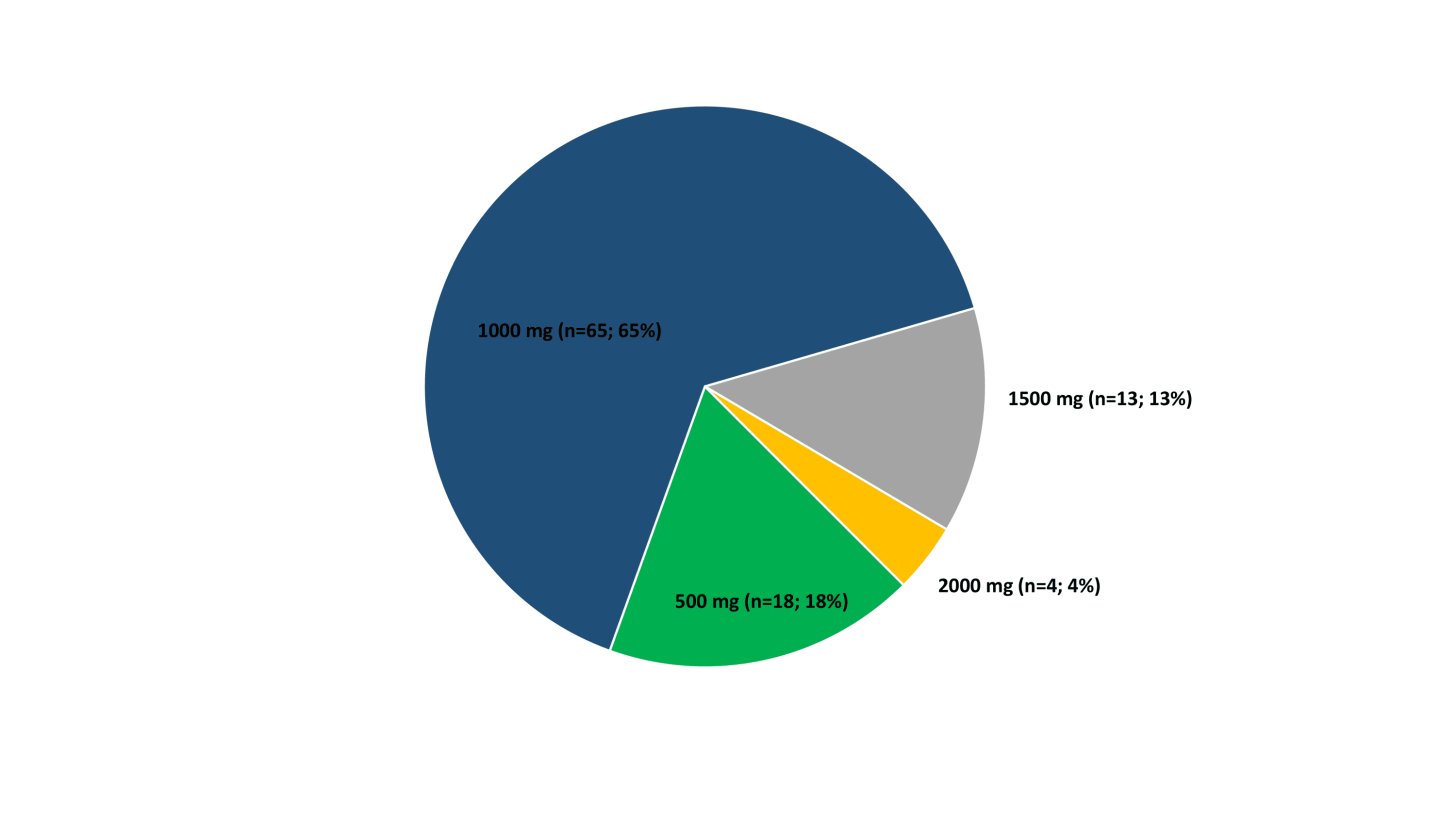
Distribution of patients according to prescribed imeglimin dose (n=100)

The study included 100 patients comprising 55 females (55%) and 45 males (45%). The mean age of the study population was 57.69 ± 9.24 years. The mean duration of T2DM was 10.38 ± 5.69 years. The mean serum creatinine was 0.86 ± 0.20 mg/dL. Patients received imeglimin at 500 mg (18%), 1000 mg (65%), 1500 mg (13%), and 2000 mg (4%) (Figure 1). Table 1 summarizes the baseline characteristics of the study population.

**Table 1:** Baseline characteristics of the study population (n=100) Data are presented as mean ± SD or n (%). SD, standard deviation; T2DM, type 2 diabetes mellitus.

Variable	Value
Age (years)	57.69 ± 9.24
Female sex	55 (55%)
Male sex	45 (45%)
Duration of T2DM (years)	10.38 ± 5.69
Serum creatinine (mg/dL)	0.86 ± 0.20
Imeglimin 500 mg	18 (18%)
Imeglimin 1000 mg	65 (65%)
Imeglimin 1500 mg	13 (13%)
Imeglimin 2000 mg	4 (4%)

Glycemic outcomes

HbA1c decreased significantly from a baseline of 7.96 ± 1.56% to 7.35 ± 1.02%, with a mean reduction of 0.62 ± 1.05% (7.8% relative reduction; p<0.001; n=77) after three months of imeglimin therapy. FPG decreased from 156.63 ± 48.48 mg/dL to 124.45 ± 20.46 mg/dL, with a mean reduction of 32.18 ± 47.02 mg/dL (20.54% relative reduction; p<0.001; n=100). PPG decreased from 217.85 ± 84.08 mg/dL to 173.22 ± 39.71 mg/dL, with a mean reduction of 44.63 ± 68.44 mg/dL (20.48% relative reduction; p<0.001; n=96). Table 2 presents the glycemic outcomes at baseline and three months.

**Table 2:** Glycemic outcomes at baseline and three months Data are presented as mean ± SD. *Baseline HbA1c values did not differ significantly between patients with and without available three-month HbA1c measurements (p<0.001), suggesting the subgroup is representative. FPG, fasting plasma glucose; HbA1c, glycated hemoglobin; SD, standard deviation; PPG, postprandial plasma glucose.

Parameter	n	Baseline (mean ± SD)	Three months (mean ± SD)	Change (mean ± SD)	t-value	p-value
*HbA1c (%)	77	7.96 ± 1.56	7.35 ± 1.02	−0.62 ± 1.05	-5.181	<0.001
FPG (mg/dL)	100	156.63 ± 48.48	124.45 ± 20.46	−32.18 ± 47.02	-6.844	<0.001
PPG (mg/dL)	96	217.85 ± 84.08	173.22 ± 39.71	−44.63 ± 68.44	-6.389	<0.001

Anthropometric, lipid, and renal outcomes

Body weight demonstrated a significant reduction from 65.70 ± 11.50 kg to 64.89 ± 11.13 kg, with a mean reduction of −0.81 ± 2.70 kg (p<0.001).

**Figure 2:**
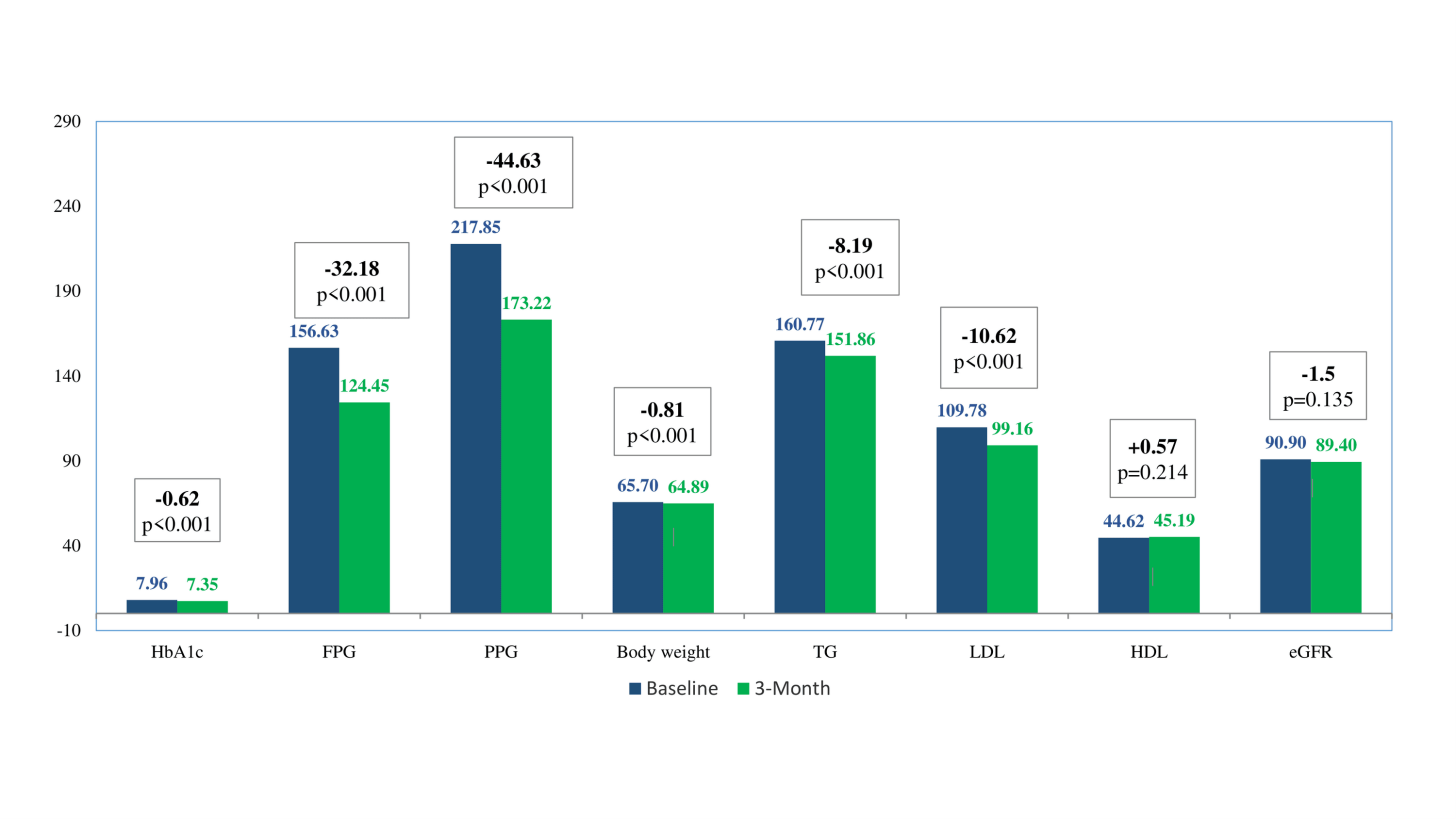
Comparative graphical presentation of glycemic, anthropometric, lipid, and renal parameters Values are represented in mean and absolute mean changes with p-values. eGFR, estimated glomerular filtration rate; FPG, fasting plasma glucose; HbA1c, glycated hemoglobin; HDL, high-density lipoprotein; LDL, low-density lipoprotein; PPG, postprandial plasma glucose; TG, triglyceride.

TGs reduced from 160.77 ± 72.42 mg/dL to 151.86 ± 58.64 mg/dL (mean reduction: −8.91 ± 17.53 mg/dL; p<0.001). LDL-C decreased significantly from 109.78 ± 30.32 mg/dL to 99.16 ± 16.85 mg/dL (mean reduction: −10.62 ± 17.32 mg/dL; 9.7% relative reduction; p<0.001). The improvement of +0.57 ± 3.77 mg/dL (p=0.214) in HDL-C was non-significant. Also, renal function, measured by eGFR, remained stable with no statistically significant change (−1.50 ± 16.59 mL/min/1.73 m²; p=0.135). The anthropometric, lipid, and renal outcomes at baseline and three months are shown in Table 3 and Figure 2.

**Table 3:** Anthropometric, lipid, and renal outcomes at baseline and three months Data are presented as mean ± SD. Bold p-values indicate statistical significance (p<0.05). eGFR, estimated glomerular filtration rate; HDL, high-density lipoprotein; LDL, low-density lipoprotein; TG, triglyceride.

Parameter	n	Baseline (mean ± SD)	Three months (mean ± SD)	Change (mean ± SD)	t-value	p-value
Body weight (kg)	99	65.70 ± 11.50	64.89 ± 11.13	−0.81 ± 2.70	-2.985	<0.001
TG (mg/dL)	67	160.77 ± 72.42	151.86 ± 58.64	−8.91 ± 17.53	-4.16	<0.001
LDL (mg/dL)	68	109.78 ± 30.32	99.16 ± 16.85	−10.62 ± 17.32	-5.056	<0.001
HDL (mg/dL)	68	44.62 ± 12.45	45.19 ± 10.16	+0.57 ± 3.77	+1.247	0.214
eGFR (mL/min/1.73 m²)	100	90.90 ± 22.08	89.40 ± 19.42	−1.50 ± 16.59	-0.904	0.135

Confounding adjustment and sensitivity analyses

To assess whether the observed glycemic improvements could be attributed to changes in background antidiabetic therapy rather than to imeglimin itself, a multivariate linear regression analysis was conducted with the change in HbA1c (∆HbA1c, three-month minus baseline) as the dependent variable. The following pre-specified covariates were entered simultaneously as independent variables: baseline HbA1c (%), age (years), sex, duration of T2DM (years), baseline body weight (kg), imeglimin dose (mg/day), and number of concomitant antidiabetic agents. The analysis was performed on 76 patients with complete data for all covariates. The number of concomitant OADs was not an independent predictor of HbA1c change (β = −0.028; 95% CI: −0.162 to 0.106; p=0.682), confirming that background polytherapy did not drive the observed glycemic benefit. In contrast, baseline HbA1c was the dominant predictor of HbA1c change (β = −0.476; 95% CI: −0.593 to −0.358; p<0.001), reflecting regression to the mean and imeglimin’s glucose-dependent mechanism. The overall model was statistically significant (R² = 0.605; adjusted R² = 0.564; F (7,68) = 14.86; p<0.001). The full multivariate regression model output is presented in Table 4.

**Table 4:** Multivariate linear regression analysis: predictors of change in HbA1c at three months (n=76) Bold p-value indicates statistical significance (p<0.05). Overall model: R² = 0.605, adjusted R² = 0.564, F (7,68) = 14.86, p<0.001. β, standardized regression coefficient; CI, confidence interval; HbA1c, glycated hemoglobin; OAD, oral antidiabetic drug; T2DM, type 2 diabetes mellitus.

Variable	β	SE	95% CI	p-value
Baseline HbA1c (%)	−0.476	0.059	(−0.593, −0.358)	<0.001
Age (years)	0.018	0.009	(−0.001, 0.037)	0.057
Sex (male)	−0.008	0.168	(−0.344, 0.328)	0.963
Duration of T2DM (years)	0.010	0.017	(−0.023, 0.043)	0.551
Baseline body weight (kg)	0.002	0.007	(−0.012, 0.017)	0.745
Imeglimin dose (mg/day)	0.000	0.000	(−0.001, 0.001)	0.909
No. of concomitant OADs	−0.028	0.067	(−0.162, 0.106)	0.682

A pre-specified sensitivity analysis was performed to further substantiate the primary findings in the context of concomitant therapy. Since only one patient in this cohort received imeglimin without any concomitant antidiabetic agent, a monotherapy-restricted sensitivity analysis was not feasible. Instead, a sensitivity analysis was conducted among the 99 patients receiving imeglimin who had at least one concomitant OAD. This represents the dominant clinical pattern in this real-world cohort of long-standing T2DM (mean disease duration: 10.38 years). In this subgroup, statistically significant and clinically meaningful reductions were confirmed across all primary glycemic endpoints: HbA1c (n=75; −0.64 ± 1.06%; t = −5.212; p<0.001), FPG (n=99; −32.44 ± 47.19 mg/dL; t = −6.841; p<0.001), and PPG (n=95; −45.34 ± 68.45 mg/dL; t = −6.457; p<0.001). The multivariate model demonstrated that the number of concomitant OADs was not an independent predictor of HbA1c change (β = −0.028; p=0.682).

Safety outcomes

Imeglimin therapy was well tolerated, with GI side effects reported in 2 (2.0%) patients, which is notably lower than that observed with metformin. Furthermore, renal function was preserved over the three-month study period, with no clinically significant change in eGFR (p=0.135). No patient required discontinuation of imeglimin during the three-month study period. Table 5 summarizes the safety profile of imeglimin.

**Table 5:** Safety profile of imeglimin therapy over three months (n=100) eGFR, estimated glomerular filtration rate; GI, gastrointestinal.

Safety parameter	n	%
GI side effects	2	2
No GI side effects	98	98
Renal function (eGFR): no significant change	100	p=0.135

## Discussion

This retrospective observational study based on real-world evidence demonstrates the efficacy and safety of imeglimin as a replacement therapy for metformin in patients with T2DM who experienced GI intolerance. The present study demonstrated statistically significant and clinically meaningful improvements in HbA1c (−0.62%), FPG (−32.18 mg/dL), and PPG (−44.63 mg/dL), along with a decrease in body weight and favorable effects on lipid levels over three months. GI side effects were reported in only 2% of imeglimin patients with no discontinuation of the therapy.

The magnitude of HbA1c reduction observed in the present study (−0.62%) is within the range reported across the pivotal TIMES trial. TIMES 1 trial demonstrated that using imeglimin 1000 mg twice daily as monotherapy reduced HbA1c by −0.87% compared with placebo over 24 weeks in Japanese patients [14]. Similarly, the long-term TIMES 2 trial reported an HbA1c reduction of 0.46% with imeglimin as monotherapy and 0.56%-0.92% with oral combination therapies over 52 weeks [15]. TIMES 3 trial observed a placebo-corrected HbA1c reduction of 0.60% when imeglimin was added to insulin at 16 weeks [16].

Complementing these pivotal trial data, the large-scale Indian real-world PRISM study reported a mean HbA1c reduction of 1.26% at six months in 722 metformin-intolerant patients, a greater magnitude compared with the present study (−0.62% at three months), attributable to the longer follow-up duration (six vs. three months) and a higher mean baseline HbA1c (8.5% vs. 7.96% in this cohort) [18]. Critically, imeglimin 1000 mg once daily was the predominant dosing strategy in the PRISM study, corroborating the dose-equivalence rationale employed in the present study and reinforcing the real-world effectiveness of imeglimin 1000 mg as a direct replacement for metformin 1000 mg in the Indian T2DM population.

The substantial reduction in FPG (−32.18 mg/dL) and PPG (−44.63 mg/dL) indicates the dual action of imeglimin. Imeglimin enhances insulin sensitivity of peripheral tissues, including skeletal muscle and hepatocytes, and also GSIS from pancreatic β-cells by targeting mitochondrial bioenergetic dysfunction [11,19]. This dual mechanism of action targeting the fundamental pathophysiological defects in T2DM provides a mechanistic basis for its effectiveness in managing both fasting and postprandial glycemic states. Specifically, imeglimin's inhibition of mitochondrial electron transport chain complex I, coupled with its reduction of mitochondrial permeability transition pore (mPTP) opening and its mitigation of oxidative stress, collectively contributes to the preservation of β-cell mass and the enhancement of insulin secretory capacity [20].

A significant observation from this study is the substantial reduction in LDL-C (−10.62 mg/dL) and TGs (−8.91 mg/dL). These findings are consistent with the pleiotropic properties of imeglimin, whose mitochondrial-targeting mechanism facilitates restoration of electron transport chain function and the reduction of oxidative stress. This effect could potentially influence lipid metabolism favorably, extending beyond its primary impact on glycemia; however, this hypothesis necessitates further investigation through focused mechanistic and clinical studies [19,21]. The neutral effect on HDL (p=0.214) is consistent with the results of the TIMES trials and the broader imeglimin meta-analysis [16,22]. The most clinically relevant outcome from this study is the GI tolerability profile. Patients with established metformin intolerance represent a clinically distinct population characterized by increased GI sensitivity, and any alternative agent presents the potential for exacerbated GI intolerance, thereby jeopardizing patient adherence. In this regard, the 2% GI side effects with imeglimin in this study, compared with the 20%-30% rate with standard metformin, are highly significant and a finding consistent with its distinct mechanism of action [6,20]. Metformin is associated with accumulation in intestinal enterocytes and has direct GI actions by stimulating the GLP-1 pathway and perturbing the microbiome, whereas imeglimin acts on mitochondria in pancreatic β-cells, liver, and skeletal muscle, with no known evidence of intestinal accumulation or direct enterocyte toxicity [11,23]. The systematic reviews and meta-analyses of the TIMES trials have demonstrated that imeglimin’s GI adverse events are comparable to placebo [24,25]. The 2% GI adverse events reported from this study, particularly from a GI-sensitive population, confirm that imeglimin does not replicate the intestinal burden of metformin.

The study demonstrated a statistically non-significant decrease in eGFR of 1.50 mL/min/1.73 m² (p=0.135), which indicates the renal safety of imeglimin. The safety and efficacy of dose-adjusted imeglimin in Japanese patients with advanced CKD and eGFR < 45 mL/min/1.73 m² over 52 weeks were demonstrated by the TWINKLE postmarket study, and this provides further support to the clinical applicability of imeglimin across the stages of CKD [17]. The predominantly G1-G2 CKD population in this study (mean baseline eGFR of 90.90 ± 22.08 mL/min/1.73 m², indicative of largely preserved renal function) did not show any clinically significant renal deterioration, consistent with imeglimin’s established renal safety profile.

From a mechanistic perspective, the parallels between imeglimin and metformin -- particularly their shared effects on hepatic glucose output and mitochondrial complex I inhibition -- suggest that imeglimin may be capable of replicating the core antihyperglycemic benefits of metformin in patients unable to tolerate the latter drug [19]. Nonetheless, imeglimin's additional pancreatic mechanism distinguishes it from metformin and provides incremental benefit in patients with diminished β-cell reserve, a common characteristic in patients with longer disease duration, such as those in this cohort (mean disease duration: 10.38 years).

The observed improvements in HbA1c, FPG, and PPG further support the potential utility of imeglimin as an alternative therapeutic option in patients intolerant to metformin. Imeglimin’s dual mechanism of action, involving enhancement of insulin secretion and improvement in insulin sensitivity through modulation of mitochondrial bioenergetics, provides a strong mechanistic rationale for its use in this clinical setting.

The present study has several limitations. First, an observational single-center design is prone to selection bias, and the absence of a parallel control arm limits inference that the improved glycemic parameters cannot be attributed to imeglimin alone because other stable concomitant OADs were used. It must be emphasized, however, that a placebo-controlled arm is inherently impracticable in a retrospective observational design; the incorporation of a concurrent placebo group would have required a prospective randomized study that was ethically unjustifiable for patients with established and symptomatic metformin intolerance who required an immediate therapeutic switch. Withholding effective antidiabetic therapy in exchange for a placebo in patients who are already experiencing uncontrolled glycemia following metformin withdrawal is not feasible. This constraint is explicitly recognized in regulatory guidance for real-world evidence studies [26], and observational pre-post designs with within-subject comparisons are accepted as appropriate methodologies for such clinical scenarios [27]. The within-subject (pre- vs. post-treatment) design used here serves as its own internal control by eliminating between-subject variability, and each patient serves as their own control, with pre-treatment (baseline) values compared directly against post-treatment (three months) values under an otherwise stable background therapeutic regimen [28]. The highly significant effect sizes observed (HbA1c: p<0.001, FPG: p<0.001, and PPG: p<0.001) are unlikely to be explained by a placebo response alone, particularly in a population with long-standing T2DM (mean disease duration: 10.38 years). Furthermore, the magnitude of HbA1c reduction (−0.62%) is consistent with that reported in the placebo-controlled TIMES 1 RCT (−0.87% vs. placebo) and the placebo-corrected TIMES 3 RCT (−0.60%), lending external validity to our findings. To address potential confounding by concomitant medications, multivariate linear regression confirmed that the number of concomitant OADs was not an independent predictor of HbA1c change (β = −0.028; p=0.682), while the overall model (R² = 0.605; p<0.001) confirmed the robustness of the primary findings. As nearly all patients (99/100) in this real-world cohort of long-standing T2DM were receiving combination therapy, a monotherapy-restricted sensitivity analysis was not feasible; however, the multivariate regression results provide equivalent or stronger evidence that background OAD use did not drive the glycemic outcomes. Nonetheless, unmeasured confounders such as dietary habits and physical activity levels, which were not systematically documented in medical records, represent a residual limitation inherent to any retrospective design. Second, the three-month follow-up period is adequate to evaluate the short-term glycemic response; however, it precludes assessment of long-term efficacy and durability of cardiovascular and renal outcomes. Future multicenter RCTs with longer follow-up periods are warranted to validate the present findings and to determine long-term safety, durability, and cardiovascular outcomes of imeglimin in the metformin-intolerant patient population.

## Conclusions

Imeglimin achieved statistically significant and clinically meaningful improvements in glycemic control, which includes HbA1c, FPG, and PPG, with additional significant reductions in LDL-C, TGs, and body weight over three months in patients with T2DM who were unable to tolerate metformin. Imeglimin was exceptionally well tolerated, and renal function remained stable during the study period. The replacement of metformin with an equivalent dose of imeglimin (1000 mg for 1000 mg), as supported by published Indian data, demonstrates comparable glycemic efficacy at this dose, providing a clinically meaningful and evidence-based rationale for the dosing strategy employed. These findings support imeglimin as an effective and well-tolerated replacement therapy for metformin in patients with T2DM who have GI intolerance. Nevertheless, future randomized controlled studies should be conducted to establish the long-term efficacy and safety profile of imeglimin in patients with T2DM.
